# Ethyl Acetate Extract of* Asclepias curassavica* Induced Apoptosis in Human Cancer Cells via Activating p38 and JNK MAPK Signaling Pathways

**DOI:** 10.1155/2019/9076269

**Published:** 2019-06-09

**Authors:** Xi Zheng, Ying Xu, Bei Liu, Yan Qi, Ying Zhang, Xiaosi Li, Xia Zhang, Xiaojia Pu, Siwen Li, Zhe Chen, Chunping Wan

**Affiliations:** ^1^The No. 1 Affiliated Hospital of Yunnan University of Traditional Chinese Medicine, Kunming 650021, China; ^2^School of Pharmacy, Yunnan University of Traditional Chinese Medicine, Kunming 650500, China; ^3^Zhejiang Hospital of Traditional Chinese Medicine, Zhejiang Chinese Medical University, Hangzhou 310006, China

## Abstract

*Background. Asclepias curassavica *L. (*Asclepiadaceae*), as a traditional medicinal plant, is used as treatment for tumors in traditional Chinese and Indian medical practice. However, its underlying molecular mechanisms remain largely unresolved. The current study investigated its antitumor activity and the underlying molecular mechanisms.* Method.* Cell viability was detected by a real-time cell analysis system and MTT assay. Antitumor effect of ethyl acetate extract of* Asclepias curassavica* (EAAC) on NIC-H1975 tumors* in vivo* was assessed in BALB/c-nu/nu mouse. Apoptosis was measured using Hoechst33342 staining and Annexin V/PI-staining. Apoptosis-related proteins and MAPK signaling pathways were analyzed based on Western blot assay.* Results.* EAAC exhibited the highest cytotoxic activity* in vitro* than other polar parts. Meanwhile, EAAC could inhibit sensitive cell line NIC-H1975 proliferation in a concentration-dependent and time-dependent manner. Furthermore, EAAC had a significant inhibitory effect on NIC-H1975 tumor growth in BALB/c-nu/nu mouse. NIC-H1975 cells showed obvious apoptosis characteristics after EAAC treatment. Fas, caspase family members caspase 3, caspase 9, and caspase 8 showed dose-dependent induction by EAAC treatment, with increasing PARP cleavage. Additionally, EAAC significantly downregulated antiapoptotic proteins Bcl-2, XIAP, survivin, and Mcl-1 and upregulated proapoptosis proteins Bak, Bax, as well as activation of p38 and JNK MAPK signaling pathways. Moreover, inhibiting p38 and JNK MAPK by pharmacological inhibitors abrogated EAAC-induced apoptosis.* Conclusion.* Our data indicated that EAAC exerted potent antitumor effect both* in vitro* and in* vivo* by triggering the apoptotic pathway.

## 1. Background

Cancer has threaten human life and health worldwide; in particular lung, breast, and colon cancer are the three most common cancers. Conventional chemotherapy plays a key role in the treatment of these cancers; however the results of treatment are fairly unpleasant, which drives us to develop a safe and highly effective tumor-inhibiting agent.

Many plants have been reported to possess various bioactivities, including antitumor activity.* Asclepias curassavica *L. (*Asclepiadaceae*), a traditional medicinal plant from Dai medicine in the southwestern China, is widely used as treatment against tumor, hemostatic disorders, inflammation, and pain [1]. Among these, the antitumor activity already received intensive attention from pharmaceutical researchers. However, its molecular mechanism remains unclear.

Impaired apoptosis is one of the hallmarks of cancer, which contribute to tumor progression and resistance to conventional cancer therapy [2]. Induction of cell apoptosis is a common strategy for cancer therapy. Successful apoptosis-inducing drugs lead tumor cells to die by directly turning on/off antiapoptotic pathway. Many evidences revealed turning on/off many death and survival genes is involved in this process. Fas and FasL have important roles in the external death receptor pathway; members of the Bcl-2 family, Bcl-2 and Bax, play a significant role in the mitochondrial-mediated cell death pathway [3–5]; in addition, cytochrome C releasing from mitochondria can activate caspase 9, which in turn activated executioner caspase 3 via cleavage induction [6].

In the present study, we found that ethyl acetate extract of* Asclepias curassavica *L. (EAAC) exerted potent antitumor effect both* in vitro* and* in vivo*; EAAC also induced apoptosis in NIC-H1975 cell line, which was related to suppressing the expression of survival proteins, including Bcl-2, survivin, and upregulating the expression of proapoptosis proteins, (e.g., bak). In particular, the cleavage of caspase 3, caspase 9, caspase 8, and Fas expression was significantly increased after EAAC treatment.

The mitogen-activated protein kinase (MAPK) superfamily consists of ERK, p38 kinase, and JNK which have been implicated in cell proliferation, differentiation, invasion, and apoptosis [7]. Generally, p38 and JNK contribute to the induction of apoptosis [8–10], whereas ERK pathway activation is important for cell survival. From current study, we confirmed the phosphorylation of p38 and JNK was remarkably induced by EAAC, which confirmed p38 and JNK MAPK signaling pathways were involved in EAAC induced-apoptosis.

## 2. Methods

### 2.1. Preparation of Extracts of Asclepias curassavica

The dried aerial parts of* Asclepias curassavica* (5kg) were boiled in water (30min/time, 4 times), the water broth was extracted with chloroform, ethyl acetate, n-butanol successively, and then each extract was concentrated by a rotary evaporator and preserved at 4°C.

### 2.2. Cell Line and Reagents

A549 cell line (human lung cancer cell line), Hela cell line (human cervical carcinoma cell line), SK-OV-3 cell line (human ovarian cancer), NIC-H1975 cell (human lung cancer cell line), K562 cell line (human leukemic cell line), S180 cell line (mouse sarcoma cell line), and H22 (mouse hepatoma cell line) were obtained from the ATCC (Manassas, VA, USA). SB203580 or SP600125 was purchased from Dalian Meilun Biology Co., Ltd. (Dalian, China). RPMI1640 medium was purchased from Gibco BRL, Life Technologies (USA). Fetal bovine serum (FBS) was purchased from Hyclone Laboratories (Logan, Utah, USA). The antibodies were purchased from Cell Signaling Technology. FITC-conjugated, Annexin V, and PI were purchased from BD Bioscience.

### 2.3. Experimental Animals

Immunodeficient BALB/c male mice (BALB/c-nu/nu) aged 6-8 weeks were purchased from Shanghai Laboratory Animal Center of the Chinese Academy of Sciences. All experiments were approved by Ethical Committee of No. 1 Hospital Affiliated Yunnan University of Traditional Chinese Medicine.

### 2.4. Cell Viability Assay

MTT assay was used to evaluate antitumor activity, and detailed method was performed as previously described [11, 12]. Label-free real-time detection technology was used for continuously monitoring tumor cells proliferation and viability. In brief, A549 and NIC-H1975 cells were seeded 96-well microtiter E-plate (Roche Applied Sciences), and the cells were treated with different concentrations of EAAC for 48h. The cell response to EAAC treatment was continuously monitored by xCELLigence SP system.

### 2.5. Antitumor Activity of EAAC In Vivo

BALB/c-nu/nu were injected* s.c.* into the right forelimb armpit with 4×10^6^ NIC-H1975 lung tumor cells, and the mice were then randomly divided into three groups: vehicle control group, EAAC group, and Cyclophosphamide (CTX ) group. The Cyclophosphamide group was injected* i.p.* with 10 mg/kg Cyclophosphamide, EAAC group was orally administered 100 mg/kg EAAC, and vehicle control group was perfused with the same volume of 0.9% physiological saline. 21 days after the last drug administration, the mice were sacrificed, then tumor volume and weight were calculated.

### 2.6. Assessment of Apoptosis

Apoptosis was measured by Annexin V/PI-staining and Hoechst 33342 staining according to the method described previously [13] For Annexin V/ PI-staining assay, NIC-H1975 cells were harvested after EAAC or MAPK inhibitor treatment, and the cells were stained with FITC-conjugated Annexin V and PI for 15 min and then analyzed with a flow cytometer (FACSCanto™II; BD Biosciences). For Hoechst dye staining, NIC-H1975 cells were stained with Hoechst33342 for 10 min after 24h EAAC treatment and were then photographed by fluorescent microscope.

### 2.7. Western Blotting Assay

Western blot was performed according to the procedures previously described [14]. Briefly, NIC-H1975 cells were treated with different concentrations of EAAC, and whole cell lysates were prepared. Proteins were electrophoresed through 10% SDS-PAGE gel and transferred to the nitrocellulose membrane. The blots were blocked with 5% BSA-TBST buffer and then incubated overnight at 4°C with primary antibodies. Membranes were washed and incubated with HRP-conjugated secondary antibody, and immune complexes were detected by enhanced chemiluminescence.

### 2.8. Statistical Analysis

Data were expressed as Mean±SD of experiments. Student's t-test was used to determine significance between two groups.* P*< 0.05 was considered to be statistically significant.

## 3. Result

### 3.1. Ethyl Acetate Extract of* Asclepias curassavica *Exerted Best Antitumor Activity* In Vitro*

Cytotoxic activity of different extract of* Asclepias curassavica* was evaluated against human lung cancer cell line A549 and NIC-H1975, human cervical carcinoma Hela, human ovarian cancer SK-OV-3, and human leukemic cell line K562 by MTT assay, using cisplatin as the reference drug. The biological results of different extract of* Asclepias curassavica* were summarized in Table 1.

As shown in Table 1, different extracts of* Asclepias curassavica* display significant antitumor activities against a panel of human tumor cell lines, among which ethyl acetate extract of* Asclepias curassavica* possessed the highest cytotoxic activity* in vitro* (Table 1). The half maximal inhibitory concentration (IC_50_) of A549, Hela, SK-OV-3, K562, and NIC-H1975 cells was calculated to be about 0.66, 0.43, 1.92, 0.76, and 0.40 *μ*g/ml, respectively. Thus, ethyl acetate extract of* Asclepias curassavica* was chosen as representative for further investigation of the antitumor activity and exploration of the underlying mechanism.

### 3.2. EAAC Caused Cytotoxicity in Dose-Dependent and Time-Dependent Manner

**Figure 1:**
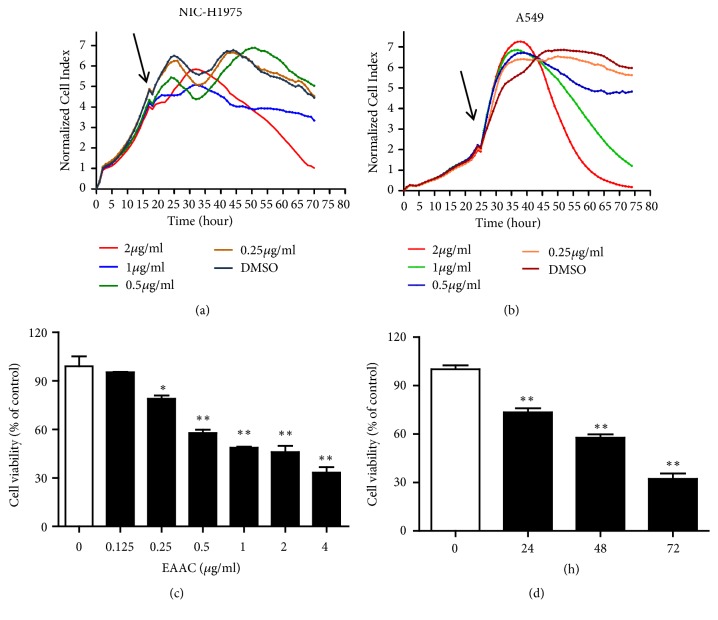
*EAAC caused cytotoxicity in a dose-dependent manner and in a time-dependent manner.* A549 and NIC-H1975 cells were seeded into 96-well microtiter E-plate. Then, these cells were treated with different concentrations of EAAC. The cell response to EAAC treatment was continuously monitored by xCELLigence SP system for 48h (Figures 1(a) and 1(b)). NIC-H1975 cells were seeded into 96-well microtiter plates, the cells were treated with various concentrations of EAAC for 48h, and cell viability was determined using a MTT assay (Figure 1(c)). NIC-H1975 cells were treated with (0.5*μ*g/ml) EAAC for the indicated times and cell viability was analyzed using a MTT assay (Figure 1(d)). Results presented mean ± s.e.m., n = 3. *∗ P* < 0.05, *∗∗ P* < 0.01 versus control group.

To further explore therapeutic potential of EAAC, the antitumor activity of EAAC was investigated by a real-time cell analysis system (xCELLigence) monitoring A549, and NIC-H1975 cell lines. Unlike the conventional endpoint cell viability assays, the real-time cell detection system continuously monitors live cells in response to the exposure of EAAC and provides kinetic cell response information from the same cell population. The results showed that dose- and time-dependent cell responses to EAAC were clearly shown in A549 and NIC-H1975 cell lines (Figures 1(a)–1(c)). Consistent with the real-time cell detection system, EAAC caused cytotoxicity against A549 and NIC-H1975 in a dose-dependent manner. Based on the IC_50_, NIC-H1975 cells were treated with 0.5 *μ*g/ml EAAC for 0, 24, 48, and 72h, and the results exhibited that EAAC exerted strong cytotoxicity against NIC-H1975 in a time-dependent manner (Figure 1(d)).

### 3.3. EAAC Exerts Significant Antitumor Effect on NIC-H1975 Tumors in BALB/c-nu/nu Mouse

**Figure 2:**
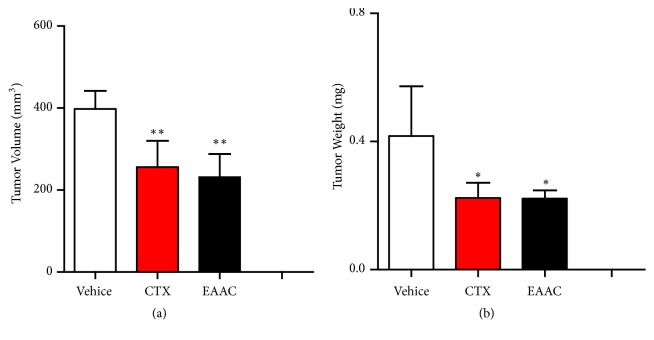
*EAAC exerts significant antitumoral effect on* NIC-H1975 tumors in BALB/c-nu/nu mouse. Nude mice (BALB/c nu/nu) were injected subcutaneously into the right forelimb armpit with 4×10^6^ NIC-H1975 lung tumor cells, 24h after injected tumor, and EAAC or vehicle was administered via intragastric administration. The experiment was terminated after 3 weeks, the mice were sacrificed, then tumor volume (Figure 2(a)) and tumor weight (Figure 2(b)) were calculated. Results presented mean ± s.e.m., n = 5. *∗ P* < 0.05, *∗∗ P* < 0.01 versus vehicle group.

EAAC showed significant antitumor activity* in vitro* as described above. In order to further evaluate* in vivo *antitumor effect of EAAC, BALB/c-nu/nu mouse model of NIC-H1975 cell was established for measuring* in vivo *antitumoral effect. Twenty four hours after injected tumor, EAAC or vehicle was administered,* i.e.*, Cyclophosphamide was injected* i.p.*, the experiment was terminated after 3 weeks, and tumor volume and weight were calculated. The result indicated that, compared with vehicle group, tumor volume and weight were lower in EAAC treated group, and tumor inhibition rates on NIC-H1975 tumors in BALB/c-nu/nu mouse were 46.82% (Figure 2), implying administration of EAAC was an effective antitumor therapy* in vivo*.

### 3.4. EAAC Induced Apoptosis in NIC-H1975 Cell Lines

**Figure 3:**
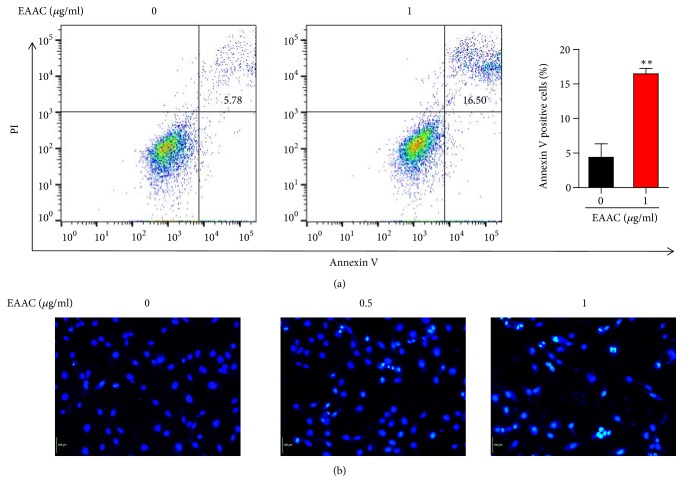
*EAAC induced apoptosis in NIC-H1975 cells.* NIC-H1975 cells were treated with various concentrations of EAAC for 24h. (a) The cells were harvested and apoptosis was measured by an Annexin V-FITC/PI. (b) The cells were stained with Hoechst33342 for 10 min after 24h EAAC treatment and were then photographed by fluorescent microscope. The result presented representative of three individual experiments.

In order to detect the effect of EAAC on apoptosis, we then choose the EAAC-sensitive cell line NIC-H1975 for further study. EAAC induced apoptosis was detected using Hoechst33342 staining and Annexin V/PI-staining by flow cytometry. Flow cytometry analysis showed that EAAC significantly promoted the NIC-H1975 cell into early stage of apoptosis in a dose-dependent manner, and the proportion of apoptotic cells in control group was 2.86%, while EAAC treatment significantly increased the proportion of apoptotic cells to be 16.50% (Figure 3(a)). Consistently, Hoechst33342 staining observed extensively chromatin condensation and nuclear fragmentation in NIC-H1975 cells after 1 *μ*g/ml EAAC treatment, suggesting these cells underwent apoptosis (Figure 3(b)). These results indicated that EAAC can effectively induce apoptosis in NIC-H1975 cells.

### 3.5. EAAC Treatment Regulates the Expression of Proteins Involved in Apoptosis

**Figure 4:**
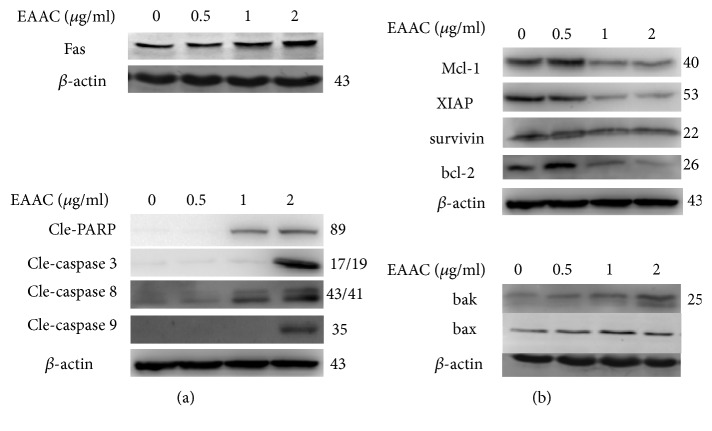
*EAAC treatment regulates the expression of proteins involved in apoptosis.* NIC-H1975 cells were exposed to different concentrations of EAAC for 48h, and whole cell extracts were obtained and determined by western blot assay using indicated antibody for assessing apoptosis-related protein. The result presented representative of three individual experiments.

To investigate the underlying mechanism of EAAC-induced apoptosis in NIC-H1975, we tested the activation of caspase 3/8/9 and consequent PARP cleavage by western blot. Data showed EAAC exerted dose-depended effect on activation of these caspases leading to increased PARP cleavage (Figure 4(a)). In addition, Fas level increased following 2 *μ*g/ml EAAC treatment (Figure 4(a)). Together, these data indicated that both mitochondrial and death receptor pathways were involved in EAAC-induced apoptosis.

 Antiapoptotic proteins are important candidates of regulating apoptosis. Bcl-2, XIAP, survivin, and Mcl-1 were checked in the induction of apoptosis in NIC-H1975 by EAAC. The result showed that EAAC significantly downregulated antiapoptotic proteins Bcl-2, XIAP, survivin, and Mcl-1. Additionally, proapoptosis proteins bak and bax were obviously upregulated after exposure to EAAC (Figure 4(b)).

### 3.6. EAAC Induced Apoptosis via Activating p38 and JNK MAPK Signaling Pathways

**Figure 5:**
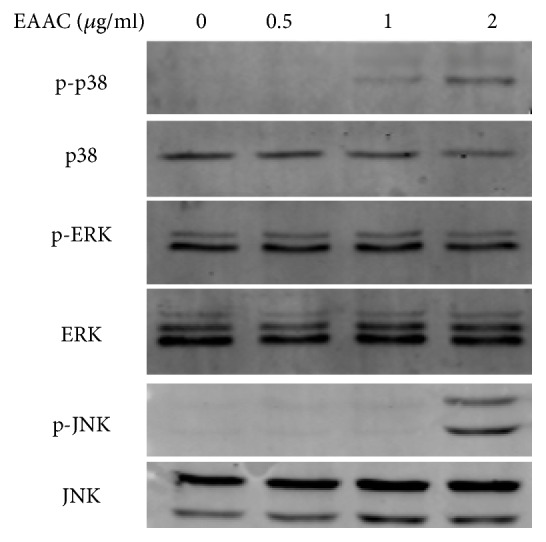
*EAAC induced apoptosis via the MAPK pathway.* NIC-H1975 cells were treated with different concentrations of EAAC, and the cell lysates were immunoblotted with pan-/phosphor-specific antibodies. The result presented representative of three individual experiments.

In order to analyze the role of p38, ERK, and JNK MAPK signaling pathways in EAAC induced cancer cells apoptosis in NIC-H1975, the phosphorylation of p38, ERK, and JNK MAPK was determined by western blot assay. NIC-H1975 cells were treated with EAAC for 24h, and then the cell lysates were immunoblotted with pan-phosphor-specific antibodies, western blot assay result showed that the phosphorylation of p38 and JNK was markedly increased after EAAC treatment, and there were no notable changes in ERK (Figure 5).

### 3.7. Inhibitor of p38 and JNK Abrogated EAAC Induced Apoptosis

**Figure 6:**
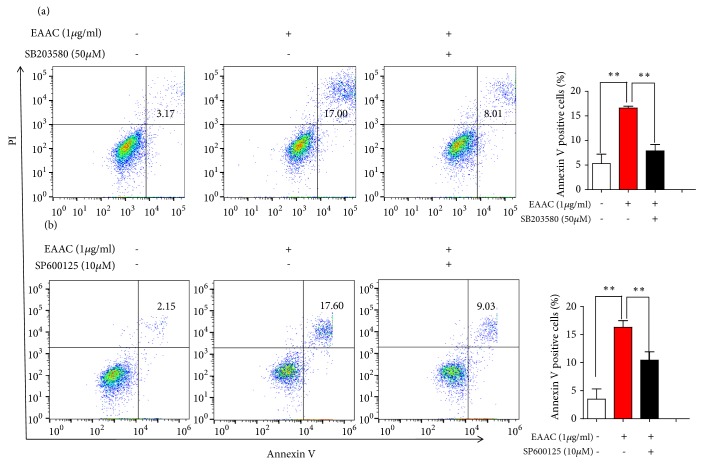
Inhibitor of p38 and JNK abrogated EAAC induced apoptosis. NIC-H1975 cells were pretreated with SB203580 or SP600125 prior to the addition of EAAC, then these cells were treated with various concentrations of EAAC for 24h. The cells were harvested and apoptosis was measured by an Annexin V-FITC/PI. Results presented mean ± s.e.m., n = 3. *∗ P* < 0.05, *∗∗ P* < 0.01 versus EAAC treated group.

EAAC treatment significantly induced the phosphorylation of p38 and JNK as described above. In order to further ensure if p38 and JNK MAPK signaling pathways were involved in EAAC induced apoptosis, cells were pretreated with SB203580 or SP600125 prior to the addition of EAAC. SB203580, an inhibitor of p38, reduced the apoptosis induced by EAAC (Figure 6(a)). The SP600125 was a specific inhibitor for JNK signaling pathway. As shown in Figure 6(b), SP600125 treatment abrogated EAAC induced apoptosis. These results are consistent with western blot results, implying that EAAC induced apoptosis through initializing the activation of p38 and JNK MAPK signaling pathway.

## 4. Discussion

Apoptosis or programmed cell death has been defined as a discrete sequence of morphological changes resulting in cell death with extensive dsDNA cleavage accompanied by chromatin compaction and segregation along the nuclear membrane [15]. Apoptosis is a central regulator of normal tissue homeostasis; it is essential for the elimination of redundant, damaged, and infected cells. In particular, apoptosis represented a fundamental antineoplastic mechanism preventing tumorigenesis of normal cells. Therefore, induction of tumor cell apoptosis was considered as an effective way of cancer therapy.

*Asclepias curassavica* L*. (Asclepiadaceae)* is a traditional medicinal plant, which was used as treatment for tumor, hemostatic disorders, inflammation, and pain in traditional Chinese medical practice; meanwhile,* curassavica* powder is also used to treat abdominal tumors in traditional Indian medicine [16–19]. Although the research about antitumor effect of* Asclepias curassavica *L. has made some progress over recent decade, their underlying molecular mechanism remains largely unknown.

In the present study, ethyl acetate extract of* Asclepias curassavica* exerted potent antitumoral effect both* in vitro* and* in vivo*. EAAC induced apoptosis was detected in NIC-H1975 cell by using flow cytometry, and the results indicated that EAAC treatment significantly promoted the NIC-H1975 cell into early stage of apoptosis with a dose-dependent manner, suggesting that EAAC exerted cytotoxicity in NIC-H1975 cell caused by inducing apoptosis.

Many chemotherapeutic agents have been thought as modulators in apoptosis-inducing signal transduction pathways, which contain the mitochondrial pathway, external death receptor pathway, and endoplasmic reticulum pathway [20]. Among them, Fas and FasL are mainly involved in the external death receptor pathway [21, 22]. Depending on the binding of Fas-associated protein to the death domain, caspase family cascade reactions are induced, which give rise to the degradation of DNA fragments and apoptosis [23]. It was considered that the expression level of Fas protein may be implied in the malignancy and development stage of tumors. Here, Fas protein expression was increased after 2*μ*g/ml EAAC treatment. Furthermore, EAAC treatment also activated caspase 3. These results indicated that EAAC induced NIC-H1975 cell apoptosis might be through upregulating the expression of caspase 3 and Fas.

It has been noted that apoptosis was regulated by the activation of the caspase family; it occurs via the intrinsic or mitochondrial pathway and the extrinsic or death receptor pathway [24, 25]. The intrinsic pathway results in cytochrome c releasing from the mitochondrial into cytosol and thus activating the initiator caspase 9. The extrinsic apoptotic pathway results from the activation of death-domain receptors and activates the initiator caspase 8. Both extrinsic and intrinsic apoptotic pathways activate executioner caspase 3 via cleavage and eventually lead to apoptosis [26, 27]. Our data showed EAAC treatment significantly induced activation of caspase 8, caspase 9, and caspase 3 at 24h in the NIC-H1975 cell, by which increase PARP cleavage. These results suggested that EAAC induced apoptosis through caspase-dependent pathway in the NIC-H1975 cell.

Many death and survival genes were involved in apoptosis, and members of the Bcl-2 family played a key role in the mitochondrial-mediated cell death pathway [28]. The proapoptotic bcl-2 family members had more increase than antiapoptotic bcl-2 family members, and the formation of pores in the outer mitochondrial membrane results in the releasing of apoptogenic mitochondrial proteins, which in turn activates caspases to induce apoptosis [29]. Our study revealed EAAC significantly downregulated antiapoptotic protein Bcl-2, XIAP, survivin, and Mcl-1. More importantly, EAAC treatment upregulated the proapoptotic protein bak.. The finding indicated that EAAC acts in both intrinsic and extrinsic apoptotic pathways via the regulation of antiapoptotic proteins and proapoptotic proteins. MAPK superfamily has implication in cell proliferation, differentiation, invasion, and apoptosis [7, 30, 31]. The MAPK signaling pathway could be activated by diverse extracellular stimuli and has a central role in cell apoptosis [32–34]. Extensive studies have shown that activation of ERK by compounds generates antiproliferative effects such as apoptosis, senescence in cancer cells, by which ERK activate apoptotic enzymes or phosphorylate transcription factors to regulate the expression of proapoptotic genes [35, 36]. JNK is a key regulator of apoptosis [10]. JNK activation contributes to TNF-*α* and UV-induced apoptosis, but it can suppress apoptosis phosphorylation of the proapoptotic Bcl-2 family protein BAD. Thus, in the regulation of apoptosis, JNK serves as double-edged sword, depending on cell type, nature of the death stimulus, duration of its activation, and the activity of other signaling pathways [37]. In this study, phosphorylation of p38 and JNK was remarkably induced by EAAC; however, there were no notable changes in ERK1/2. Moreover, we observed that inhibition of p38 and JNK MAPK by pharmacological inhibitors (SB203580 and SP600125) abrogated EAAC-induced apoptosis, suggesting EAAC induced apoptosis through regulation of p38 MAPK and JNK signaling pathways.

In this study, we establish that EAAC had potent antitumor effect both* in vitro* and* in vivo* by triggering cell apoptotic pathway. These data paved the way for further evaluation of EAAC as a new anticancer herb in Chinese traditional medical practice.

## Figures and Tables

**Table 1:** IC_50_ values of different extract of *Asclepias curassavica* in different types of tumor cell lines after 48h of treatment.

Component	Cell lines (IC_50_, *μ*g/mL) ^*a*,*b*^				
	A549	Hela	SK-OV-3	K562	NIC-H1975
Water extract	14.72	1.96	21.97	113.00	43.63
Chloroform extract	1.85	4.91	5.92	3.45	1.75
Ethyl acetate extract	0.66	0.43	1.92	0.76	0.40
Cisplatin	11.54	20.52	12.44	8.95	5.43

^*a*^Cytotoxicity as IC_50_ values for each cell line, the concentration of compound that inhibits 50% of the cell growth measured by MTT assay.

^*b*^Each value was reproduced in triplicate.

## Data Availability

The data used to support the findings of this study are available from the corresponding author upon request.
